# Management of a One-wall Intrabony Osseous Defect with Combination of Platelet Rich Plasma and Demineralized Bone Matrix- a Two-year Follow up Case Report

**Published:** 2015-09

**Authors:** Parthasaradhi Thakkalapati, Chitraa R Chandran, Aravindhan Thiruputkuzhi Ranganathan, Ashish Ratahanchand Jain, Priya Prabhakar, Suganya Padmanaban

**Affiliations:** 1 Dept. of Periodontics, Tagore Dental College and Hospitals, Chennai, India.; 2 Dept. of Prosthodontics, Tagore Dental College and Hospitals, Chennai, India

**Keywords:** Demineralized bone matrix, Intrabony Defect, Platelet Rich plasma

## Abstract

Periodontal regeneration in a one-wall intrabony defect is a challenging and complex phenomenon. The combination therapy of commercially available bone grafts with the innovative tissue engineering strategy, the platelet rich plasma, has emerged as a promising grafting modality for two and three walled intrabony osseous defects. The application of this combination approach was attempted in a most challenging one-wall intrabony defect. Open flap debridement and placement of combination of autologous platelet rich plasma(PRP) and demineralized bone matrix was done in one-wall intrabony defect in relation to tooth #21 in a 30 year old female patient. The 6-month follow- up results showed significant improvement in clinical parameters. Radiographic evidence of bone formation was observed as early as 3 months with almost complete fill by 6 months post-operatively. The results were maintained over a period of 2 years.

## Introduction

Periodontal regeneration is defined histologically as regeneration of the tooth’s supporting tissues, including alveolar bone, periodontal ligament, and cementum over previously diseased root surface.[1] Periodontal regeneration in intrabony defects has been successfully attempted with a variety of different approaches. Hemiseptal defects i.e., vertical defects in the presence of adjacent roots and where half of a septum remains on the tooth, represents a special case of one-wall defects and the treatment is always a challenge despite the various periodontal regenerative therapies. 

Regenerative therapy is strongly superior when compared to open flap debridement alone and a wide array of graft materials have been applied and evaluated clinically, including autogenous bone grafts, allografts and alloplasts.[2] Demineralized freeze-dried bone allograft (DFDBA), which is shown to be both osteoconductive and osteoinductive, has been used alone and in combination with other treatment modalities for periodontal therapy.[3] Histologic evidence of periodontal regeneration, including new bone, periodontal ligament and cementum formation has been reported for demineralized freeze-dried bone allografts.[4]

The platelet rich plasma (PRP) is an innovative tissue engineering strategy in boosting the periodontal wound healing and periodontal regeneration.[5] The growth factors within PRP like platelet derived growth factor (PDGFaa, PDGFbb, and PDGFab), transforming growth factor beta (TGF-β1, TGFβ2), vascular endothelial growth factor (VEGF), epithelial growth factor (EGF), and insulin-like growth factor (IGF-1) produce a multitude of effects. PDGF is a potent mitogenic and chemotactic factor for both fibroblasts and osteoblasts. In vivo studies have shown PDGF to stimulate bone formation and consistently enhance wound fill.[6] TGF stimulates the proliferation of osteoblast precursor cells, has a direct stimulatory effect on bone collagen synthesis, and also decreases bone resorption by inducing apoptosis of osteoclasts.[7]

Many studies have shown that the combination of PRP with demineralized bone matrix is effective in treating periodontal two and three walled intrabony defects.[8-11]

The aim of present study is to assess the effectiveness of a combined regenerative therapy consisting of bone graft and PRP in a clinically challenging one-wall intrabony defect.

## Case Report

**Figure 1:**
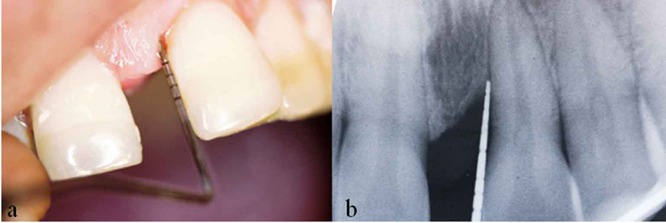
a: Pre-operative view of maxillary left central incisor shows 6mm pocket depth on mesial aspect b: Pre-operative radiographic view of maxillary left central incisor shows 5mm of intrabony defect

With the chief complaint of increase in spacing between upper front teeth for the past 6 months, a 30-year-old systemically healthy female reported to the Department of Periodontics, Tagore Dental College and Hospital, Chennai, India. On examination, there was 6mm probing pocket depth (PPD) in mesial aspect of tooth# 21, 6.5mm of clinical attachment level (CAL) with Miller’s grade I mobility (Figure 1a) and mild signs of inflammation were noticed. The teeth involved were found to be vital. A periapical radiograph showed interdental angular bone loss mesial to tooth#21 with a defect depth more than 4mm and a defect angle of 37^o^ (Figure 1b). The treatment plan was to perform open flap debridement and employ combination of PRP and DFDBA matrix to fill the one wall defect. 

Initial phase-I therapy was done with scaling, root planning and oral hygiene instructions. Splinting was done in relation to teeth # 21, 22 and 23. Two weeks after phase-I therapy, there was resolution of inflammation but the probing depth was still 6mm in the mesial aspect of tooth #21. Informed patient consent was obtained and the patient was scheduled for flap surgery.

**Figure 2:**
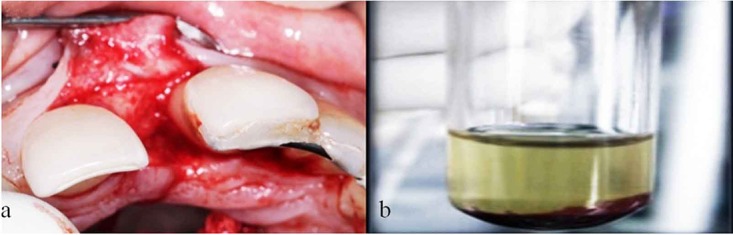
a: Following elevation of f  ull thickness flap and degranulation of interproximal area, one-wall intrabony defect mesial to tooth # 21 is revealed.  b: Freshly-prepared platelet rich plasma.

**Figure 3:**
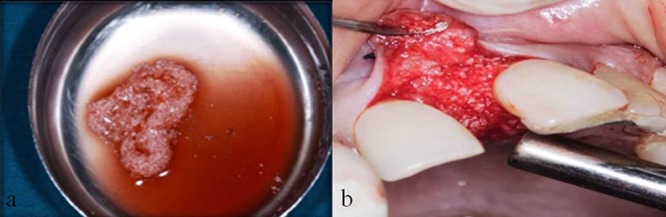
a: DFDBA combined with coagulated PRP prior to insertion into defect  b: Defect on the mesial aspect of tooth #21 grafted with DFDBA /coagulated PRP mixture

The surgical procedure was performed by local anesthesia. Mucoperiosteal flaps were reflected using papilla preservation flap technique involving the region of teeth #11, 21. A one-wall intrabony defect was exposed mesial to tooth # 21 (Figure 2a) and surgically 4.5 mm infrabony defect was evaluated. The area was thoroughly debrided and root planning was performed. Briefly, prior to surgery, PRP was prepared following the protocol described by Marx and Garg in 2005.[12] 10ml intravenous blood collected through a venipuncture in the antecubital vein was transferred to a test tube containing 1 ml of 10% trisodium citrate anticoagulant solution and centrifuged at 1200 rpm for 20 minutes, which resulted in two fractions. The plasma along with the top 2ml of red blood cells was again centrifuged at 2000 rpm for 15 minutes to get three basic fractions, platelet-poor plasma (PPP) at the top of the preparation (supernatant), platelet rich plasma (PRP) in the middle and the red blood cell fraction at the bottom. The top 80% fraction corresponding to PPP was aspirated with a pipette, leaving the residual (0.5-2ml) platelet concentrate (Figure 2b). Then the coagulation of platelet rich plasma was obtained by adding 1ml Batroxobin (Pentapharm) and 1ml of 10% calcium gluconate (Medicos pharma). Within a few seconds, a sticky gel consistency was obtained to be mixed with the bone graft and applied to the surgical site (Figure 3a).

**Figure 4:**
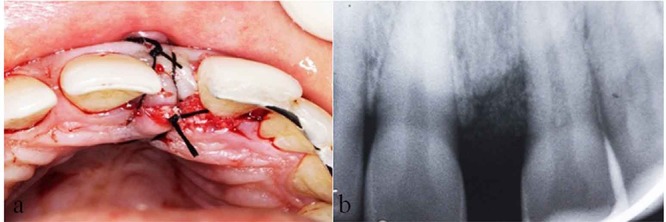
a: Flaps sutured with No.3-0 silk.  b: Post operative immediate radiographic view.

The mixture of coagulated PRP with demineralized bone matrix was tightly condensed in to the intrabony defect (Figure 3b) followed by repositioning of mucoperiosteal flaps by simple interrupted sutures. (Figure 4) and the periodontal dressing was placed. Antibiotics and analgesics were prescribed (Amoxicillin 500mg every 6 hours for 7 days and Aceclofenac 100mg every 12 hours for 3 days) and Chlorhexidine Digluconate rinses (0.2%) was recommended as an adjunctive therapy for 2 weeks. 

One week post operatively, dressings and suture removal was done. Mechanical oral hygiene was initiated by the patient. Patient was examined weekly up to 1 month after surgery and then at 3, 6 months up to 2 years. Proper Supportive periodontal therapy was monitored.

## Results

The healing was uneventful, indicating biocompatibility of both grafting modalities. The patient showed good compliance and satisfactory oral hygiene maintenance during the course of observation period.

**Figure 5:**
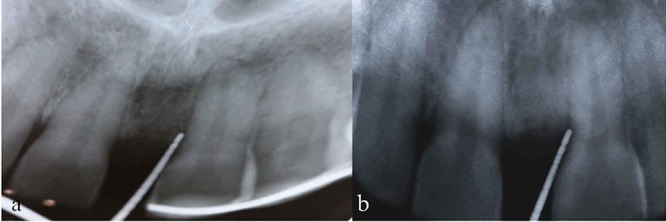
a: Post-operative radiographic view of IOPA at 3rd month  b: Post-operative radiographic view of IOPA at 6th month

**Figure 6:**
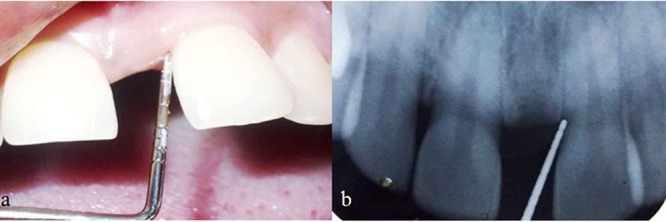
a: Clinical healing of treated defect 2 years postoperative showing 0.5mm probing depth only.  b: 2 years postoperative radiographic view of tooth #21 region

At 6 months postoperatively in the region of tooth#21, clinical examination of the treated one wall intrabony defect showed significant probing depth reduction and clinical attachment gain compared to baseline values. The probing depth reduced to 0.5mm and gain in the clinical attachment levels was 6.5mm with no mobility or bleeding on probing. The radiographs also showed good bony fill which was almost complete by 6 months (Figure 5). The results were maintained up to the 2-year follow up period (Figure 6).

## Discussion

Regeneration of tooth supporting structures destroyed by periodontitis is a major goal of periodontal therapy and is perhaps one of the most complex phenomenons to occur.

With respect to the treatment of intrabony defects, the results of meta- analysis conclude that bone grafts increase bone level, reduce bone loss, increase clinical attachment level, and reduce probing pocket depths when compared to open flap debridement procedures.[2]

This report is mainly concerned with the healing of one-wall intraosseous bony defect of more than 4mm depth and 37^o^ defect angle. Though there are indications that two and three walled bony defects respond better to regenerative therapy than one-wall defects, several studies have demonstrated that the extent of vertical attachment gain[13-14] or osseous filling[13, 15-16] correlates with the total corono-apical extent of the bony defects, including the one-wall component. In other words, the deeper the bony defect (>4mm),[18-19] the greater attachment gains and the better osseous filling may be expected. 

Another aspect of infra-alveolar defects is the defect angle. Narrow defects allow for better gains of attachment and bone substance than wide defects. Varying threshold values were used to define narrow defects in different studies; < 26° by Klein *et al.* in 2001,[15] ^<^ 37° by Eickholz *et al.* in 2004[16] and ≤ 22° by Tsitoura *et al.* in 2004.[17] The DFDBA and PRP combination is more effective for the treatment of infrabony defects in terms of amount of CAL gain, PPD reduction, and bone fill.[9]

PRP, as used in this study, may affect the wound healing not only by a release of PGFs from platelets, but also because of other physical and chemical properties. The PRP preparation because of its high fibrin content, presents a sticky characteristic that works as a hemostatic and stabilizing agent and may aid blood clot and bone graft immobilization in the defect area.

Blood clot immobilization has been suggested as an important event in the early phases of wound healing in periodontal regenerative procedures.[18]

Clinical studies evaluating the combination of PRP and DFDBA showed successful periodontal regeneration in two and three wall intrabony defects.[8-11] A histologic study by Jung seok lee *et al.*,[19] in a one-wall intrabony defect showed that customized n-HA block could provide the space for periodontal tissue engineering with minimal inflammation.

In this case, the DFDBA graft with PRP was sufficient to fill the one wall intrabony defect with predictable healing and improvement in clinical parameters and the results were maintained for a longer period.

## Conclusion

Within the limits of present case report, it can be concluded that the combination therapy of platelet rich plasma (PRP) with demineralized bone grafts holds a promising potential for CAL gain, PPD reduction, and bone fill even in a one-wall intrabony defect. 

However, further long term clinical research with larger sample size and confirmatory histological evaluations and advanced radiodiagnostical assessment can provide a greater insight to better assess the clinical benefits and actual regenerative process of the combination approach using PRP with bone graft.
